# Micro-HCCs in rats with liver cirrhosis: paradoxical targeting effects with vascular disrupting agent CA4P

**DOI:** 10.18632/oncotarget.19339

**Published:** 2017-07-18

**Authors:** Yewei Liu, Ting Yin, Frederik De Keyzer, Yuanbo Feng, Feng Chen, Jianjun Liu, Shaoli Song, Jie Yu, Vincent Vandecaveye, Johan Swinnen, Guy Bormans, Uwe Himmelreich, Raymond Oyen, Jian Zhang, Gang Huang, Yicheng Ni

**Affiliations:** ^1^ Biomedical Group, Campus Gasthuisberg, KU Leuven, Leuven 3000, Belgium; ^2^ Institute of Clinical Nuclear Medicine, Renji Hospital, Shanghai Jiao Tong University School of Medicine, Shanghai 200127, China; ^3^ Shanghai University of Medicine and Health Sciences, Shanghai 201318, China; ^4^ Institute of Health Sciences, Shanghai Jiao Tong University School of Medicine (SJTUSM) and Shanghai Institutes for Biological Sciences (SIBS), Chinese Academy of Sciences (CAS), Shanghai 200025, China; ^5^ Jiangsu Province Academy of Traditional Chinese Medicine, Nanjing 210028, China

**Keywords:** vascular-disrupting agents, combretastatin A4 phosphate, therapeutic response, microcancer, hepatocellular carcinoma

## Abstract

We sought to investigate anticancer efficacy of a vascular disrupting agent (VDA) combretastatin A-4 phosphate (CA4P) in relation to tumor size among hepatocellular carcinomas (HCCs) in rats using magnetic resonance imaging (MRI) and postmortem techniques. Nineteen rats with 43 chemically-induced HCCs of 2.8–20.9 mm in size on liver cirrhosis received CA4P intravenously at 10 mg/kg. Tumor-diameter was measured by T2-weighted imaging (T2WI) to define microcancers (< 5 mm) versus larger HCCs. Vascular responses and tissue necrosis were detected by diffusion-weighted imaging (DWI), contrast-enhanced T1-weighted imaging (CE-T1WI) and dynamic contrast enhanced (DCE-) MRI, which were validated by microangiography and histopathology. MRI revealed nearly complete necrosis in 5 out of 7 micro-HCCs, but diverse therapeutic necrosis in larger HCCs with a positive correlation with tumor size. Necrosis in micro-HCCs was 36.9% more than that in larger HCCs. While increased diffusion coefficient (ADC_diff_) suggested tumor necrosis, perfusion coefficient (ADC_perf_) indicated sharply decreased blood perfusion in cirrhotic liver together with a reduction in micro-HCCs. DCE revealed lowered tumor blood flow from intravascular into extravascular extracellular space (EES). Microangiography and histopathology revealed hypo- and hypervascularity in 4 and 3 micro-HCCs, massive, partial and minor degrees of tumoral necrosis in 5, 1 and 1 micro-HCCs respectively, and patchy necrotic foci in cirrhotic liver. CD34-PAS staining implicated that poorly vascularized micro-HCCs growing on liver cirrhosis tended to respond better to CA4P treatment. In this study, more complete CA4P-response occurred unexpectedly in micro-HCCs in rats, along with CA4P-induced necrotic foci in cirrhotic liver. These may help to plan clinical applications of VDAs in patients with HCCs and liver cirrhosis.

## INTRODUCTION

Combretastatin A4 phosphate (CA4P), as a Combretastatin family member initially derived from the South African willow tree *Combretum caffrum* [1], has become a leading vascular disrupting agent (VDA) for cancer therapy over the past decades [2]. CA4P takes effect as a potent and reversible tubulin depolymerizing agent to damage the existing tumor blood vessels [2, 3]. In a variety of implanted tumor models conducted in preclinical studies, CA4P induces rapid tumor vascular disruption as early as less than 1 hour resulting in extensive intratumoral necrosis within 12 hours [4, 5]. Nevertheless, VDA therapy features a viable rim consisting of layers of residual cancer cells at tumor periphery [5], subsequently leading to tumor relapse over several days [6]. This accentuates the necessity to combine CA4P treatment with other therapeutics such as chemotherapy [7], conventional radiotherapy [8], internal targeted radiotherapy [6] and antiangiogenic therapy [9, 10]. To date, the safety and efficacy of CA4P plus chemo in patients with advanced non-small cell lung cancer [11–13], anaplastic thyroid cancer [14] and platinum-resistant ovarian cancer [13] have been under evaluations in phase II/III clinical trials. However, neither animal models of implanted tumors nor advanced clinical investigations on human cancers could forecast how CA4P functions in the early stage of primary HCCs.

A positive correlation between increasing tumor volume and better therapeutic effect following VDA treatment has been noticed, since the antitumor efficacy of VDAs seemed to increase as tumors grew larger [15]. Such a correlation has been observed in multiple murine allograft and xenograft models in preclinical studies [16–18]. For instance, in the rat allograft model of subcutaneous rhabdomyosarcomas, CA4P efficacy in large tumors (≥ 14 cm^3^) was 16.6-fold stronger than that in small tumors (< 1 cm^3^) [17]. Similarly, intraperitoneal injection of ZD6126 led to nearly 90% necrosis in tumors larger than 1 g compared with only ~ 25% in the smaller ones of less than 0.3 g in several mouse xenograft models including rodent sarcoma, squamous cell carcinoma and fibrosarcoma, as well as human renal cell carcinoma, Kaposi's sarcoma and breast carcinoma [18]. Furthermore, this trend has also been implied in the clinical studies of advanced anaplastic thyroid carcinoma [19]. Despite these strong evidences, the underlying mechanisms remain to be unraveled. The inferior effects of VDAs in smaller tumors are likely due to their main portions of blood supply largely rooting from the vessels of the surrounding normal tissues [17, 18]. Indeed, tumors smaller than 5 mm in diameter often lack their own vasculature and are nourished by the nutrients diffused from their host organs [20].

As CA4P causes acute tumoral necrosis within hours, the conventional imaging criteria, Response Evaluation Criteria In Solid Tumors (RECIST) routinely adopted as the end points of VDA trials, cannot fully meet the growing needs of detecting early and transient tumor vascular reaction occurring prior to the change of tumor size [10]. Magnetic resonance imaging (MRI) is known to be of high sensitivity and excellent soft tissue contrast to identify rat liver tumors as small as 2 mm in size [21]. To date, multiparametric methods including dynamic contrast enhanced (DCE)-MRI and diffusion-weighted imaging (DWI) have been increasingly applied in both preclinical and clinical studies for acquiring functional information such as blood perfusion, fluid diffusion, blood volume, vascular permeability and extravascular extracellular space, and for noninvasively monitoring the real-time vascular responses to therapies [10, 22, 23].

**Figure 1:**
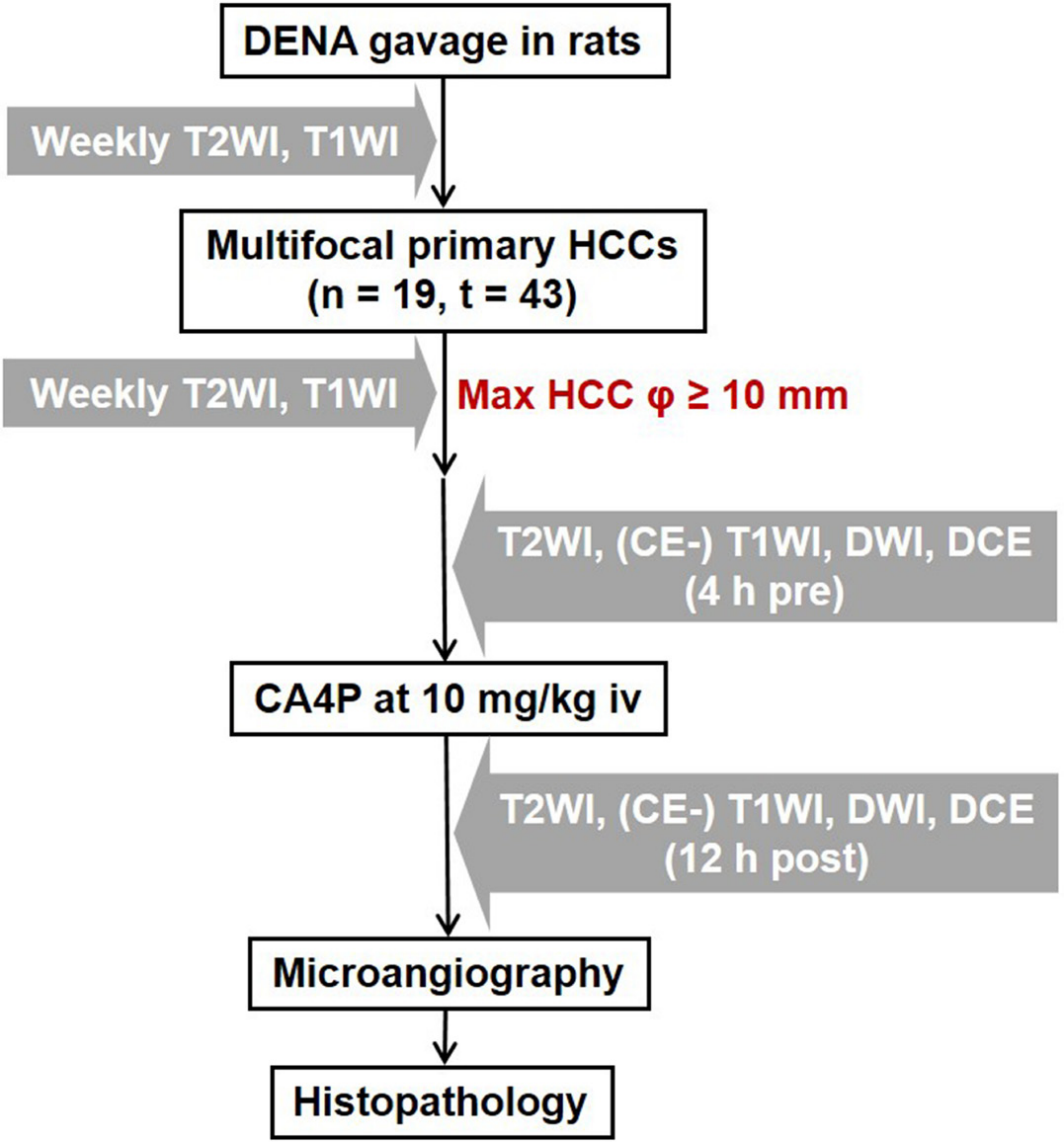
Flow chart of experimental design DENA: diethylnitrosamine; HCC: hepatocellular carcinoma; T2WI: T2-weighted imaging; CE: contrast-enhanced; T1WI: T1-weighted imaging; DWI: diffusion-weighted imaging; DCE: dynamic contrast enhanced; CA4P: combretastatin A4 phosphate; n: number of animals; t: number of tumors; Ø: diameter; h: hour(s); iv: intravenous(ly).

In the present study (Figure 1), we employed a chemically induced primary liver cancer model in rats, and evaluated the therapeutic efficacy of CA4P against HCCs in differential sizes, especially in the hepatic microcancer lesions in diameter ranging from 2 to 5 mm. Translationally, a 3.0T clinical MRI with a human wrist coil was utilized to characterize the *in vivo* early vascular responses to CA4P within 12 hours, and the imaging findings were further verified by *ex vivo* microangiography and histopathology.

## RESULTS

### General conditions

In total, 43 primary HCC lesions, superimposed on varying degrees of liver cirrhosis, were successfully generated in the 19 rats. Among them, 7 rats were identified with micro-HCCs together with other 17 larger HCCs (Table 1). All rats survived the experimental procedures including DENA gavage for hepatocarcinogenesis, gas anesthesia, MRI scanning with contrast administration, and intravenous CA4P treatment. All rats were sacrificed 12 h after CA4P treatment as the endpoint of *in vivo* study.

**Table 1:** Intra-individual comparison of CA4P-induced tumor necrosis (%) among rats with both micro-HCCs and larger HCCs

	Micro-HCCs					Larger HCCs				
	Tumor code	CA4P-induced necrosis (%)	Tumor diameter (mm)	*Tumor vascularity	**Tumor differentiation	Tumor code	CA4P-induced necrosis (%)	Tumor diameter (mm)	*Tumor vascularity	**Tumor differentiation
**Rat 1**	**Tumor 1**	93.7	2.8	+	I	#**107**	56.2	6.9	+	II
						#**105**	4.5	6.3	+++	III
**Rat 2**	**Tumor 2**	94.2	4.0	+	I	#**012**	57.4	9.0	++	III
						#**007**	47.5	5.9	+	II
**Rat 3**	**Tumor 3**	90.5	4.3	+	II	#**166**	69.4	7.6	++	III
						#**164**	46.2	6.9	++	III
						#**165**	21.8	9.7	++	III
						#**168**	16.4	6.5	++	III
						#**160**	13.1	10.2	+++	III
						#**171**	0.0	10.9	++	III
						#**163**	0.0	5.8	+	II
**Rat 4**	**Tumor 4**	96. 5	4.9	+	II	#**080**	18.8	10.3	++	III
**Rat 5**	**Tumor 5**	82.8	3.8	+++	III	#**056**	82.0	11.7	++	III
						#**054**	40.1	7.4	++++	IV
**Rat 6**	**Tumor 6**	23.2	4.1	++	III	#**049**	14.1	8.9	++	II
						#**044**	0.0	8.0	++++	IV
**Rat 7**	**Tumor 7**	1.6	4.7	++++	IV	#**133**	79.1	9.4	+	III
**Mean ± SD**		68.9 ± 39.4	4.1 ± 0.7	/	/		33.3 ± 28.3	8.4 ± 1.8	/	/

Note:

*Tumor vascularity was semi-quantificationally scored: (+) similar vascular density to the liver parenchyma; (++) dense vasculature without vascular lakes; (+++) denser vasculature with variously sized vascular lakes; (++++) full of enlarged vascular lakes. **Tumor differentiation was graded by a modified 4-scale Edmondson and Steiner system [29]: (I) highly differentiated, consisting of tumor cells of moderate size arranged in thin trabeculae; (II) larger cells with active nuclear mitosis and possible pseudoglandular structures often with steatosis; (III) larger nuclei and more hyperchromatic or increased mitotic figures, granular and acidophilic cytoplasm, often with giant tumor cells; (IV) undifferentiated tumor cells with hyperchromatic nuclei and loss of trabecular pattern often with hypervascularity and angioinvasion.

### Therapeutic efficacy enhanced with increasing tumor volume in larger HCCs

**Figure 2:**
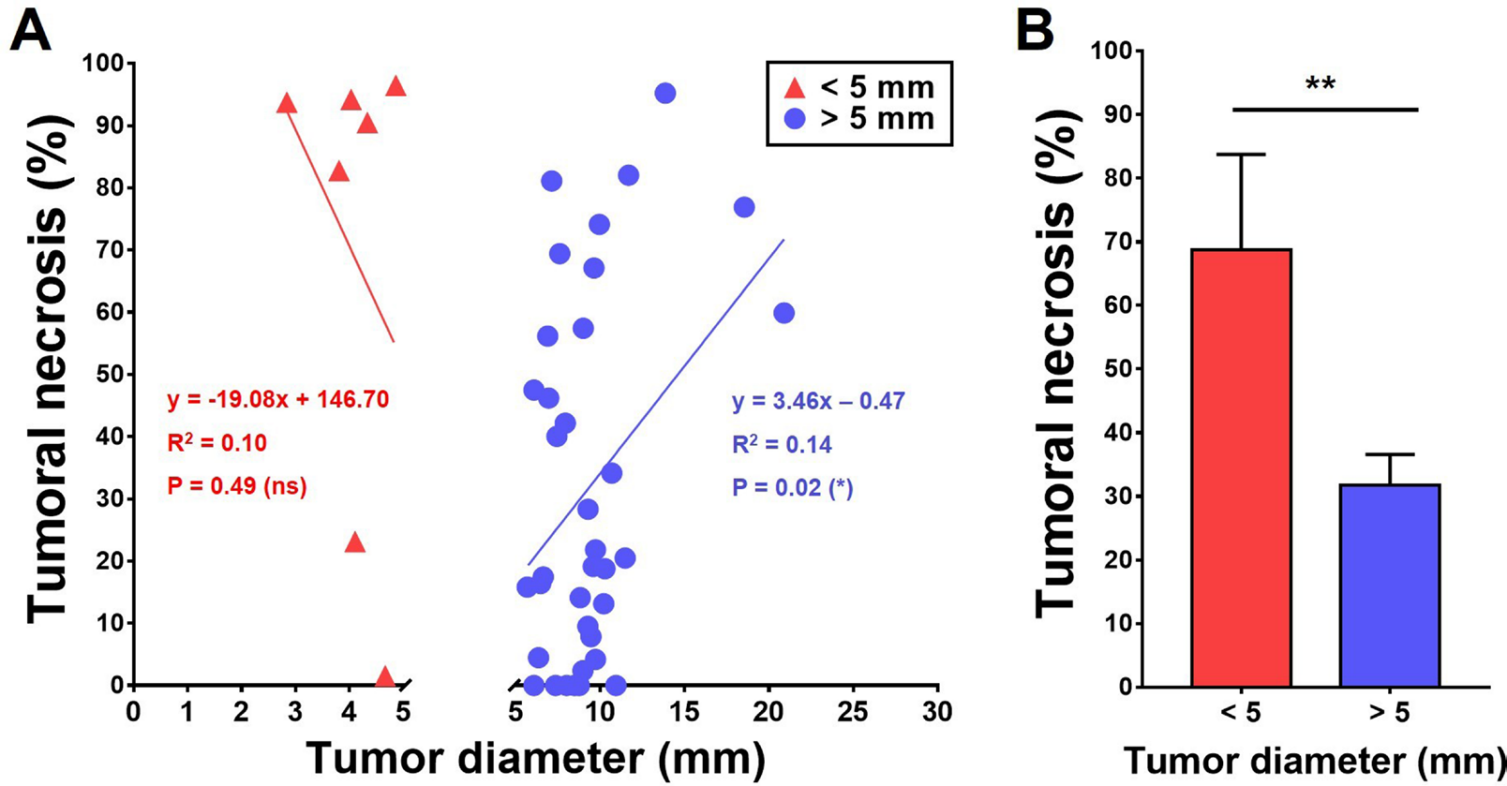
Comparison of CA4P therapeutic efficacy between micro-HCCs and larger HCCs (**A**) Scatter plots of percentage of CA4P-induced tumoral necrosis in micro-HCCs ≤ 5 mm and in HCCs > 5 mm. Significant negative linear correlation was identified between CA4P-induced tumoral necrosis and tumors with diameter > 5 mm (*P < 0.05), while tumoral necrosis was not linearly correlated with tumors with diameters of ≤ 5 mm. (**B**) Bar chart comparing the mean percentage of CA4P-induced tumoral necrosis between micro-HCCs of ≤ 5 mm and larger HCCs of > 5 mm (**P < 0.01).

We first compared CA4P-induced tumoral necrosis among 43 HCCs in various tumor diameters to investigate the relationship between antitumor efficacy of CA4P and tumor size of primary HCCs (Figure 2). In line with the previous studies that CA4P showed increased activity in larger tumors [17, 18], CA4P efficacy in this study appeared positively correlated with the larger HCCs with diameters ranging from 5.7 mm to 20.9 mm, though showing great disparities in proportion of therapeutic necrosis (Figure 2A).

### Paradoxical effects of CA4P in hepatic microcancers

Surprisingly, extensive therapeutic tumoral necrosis frequently occurred in a subgroup of smaller HCCs, namely hepatic microcancers or micro-HCCs, which were smaller than 5 mm in diameter (Figure 2A). Tumoral necrosis ranging from 80% to nearly 100% was found in 5 out of 7 microcancer lesions. Quantitatively, the rate of tumoral necrosis in the hepatic microcancers was 36.9% higher than that in the larger HCCs after CA4P treatment (Figure 2B). Intraindividual comparison of tumoral necrosis between these micro-HCCs and their larger-sized counterparts further verified this tendency (Table 1).

**Figure 3:**
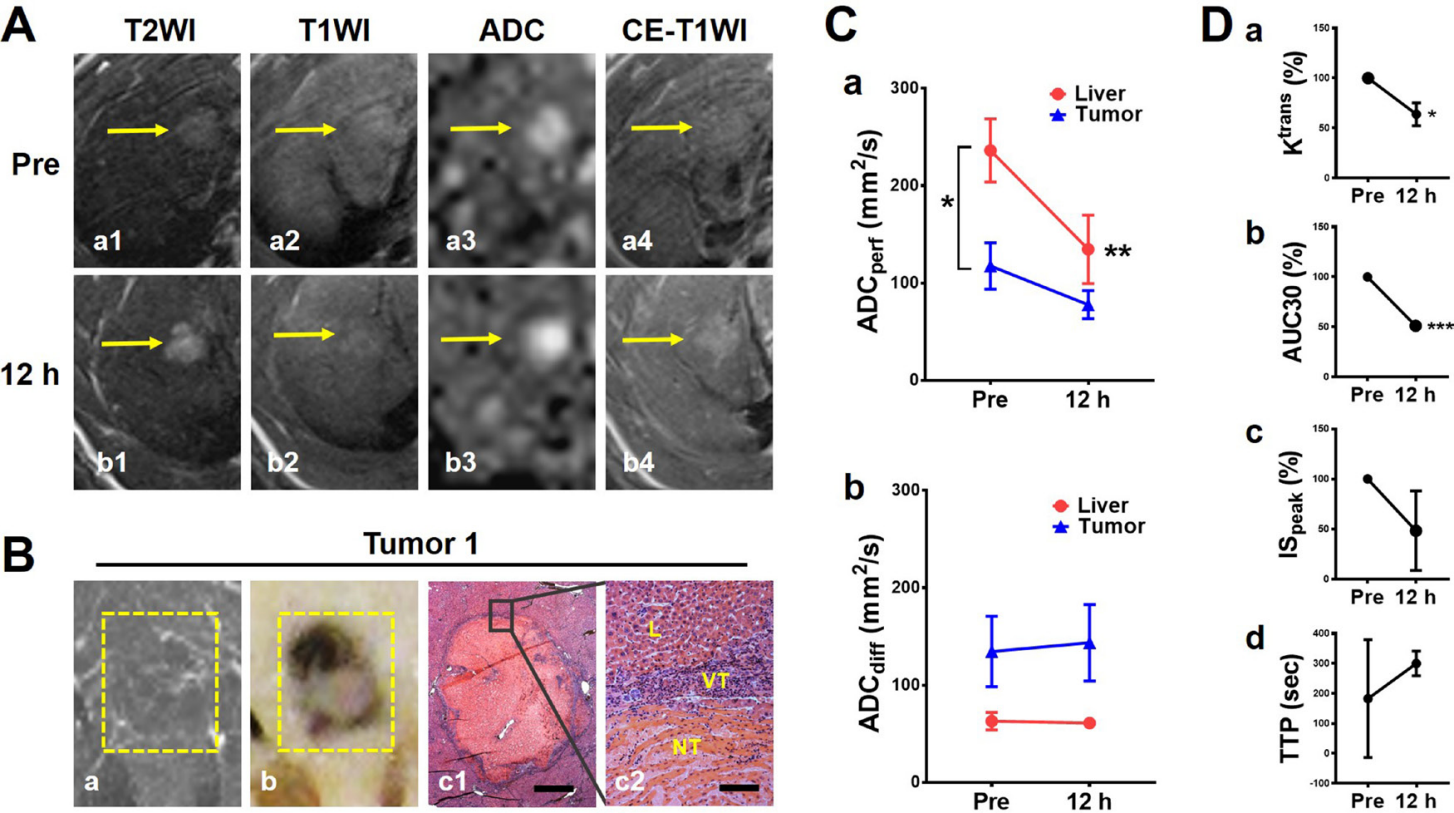
*In vivo* MRI and post-mortem verifications of a representative micro-HCC with nearly complete necrosis induced by CA4P (**A**) *In vivo* MRI findings of microcancer Tumor 1 (arrows): on T2WI, hyperintense before treatment and increased hyperintensity at 12 h (a1-b1); on precontrast T1WI, there were no obvious changes with nearly isointensity (a2-b2); on ADC map, moderate hyperintense at baseline and increased signal at 12 h (a3-b3); and on CE-T1WI, minimal enhancement at baseline and delayed contrast enhancement at 12 h after CA4P treatment (a4-c4). (**B**) Corresponding microangiography (a) depicted scarce tumor vascularity. Macrophotograph (b) and histopathology (H&E staining; c1, × 25 original magnification, scale bar = 400 μm; c2, × 200 original magnification, scale bar = 50 μm. NT: necrotic tumor; VT: viable tumor; L: liver.) revealed nearly complete intratumoral necrosis. (**C**) Quantification of ADCs derived from DWI. ADC_perf_ (a) indicated blood perfusion sharply decreased in cirrhotic liver after CA4P treatment (**P < 0.01) with a decline also in tumor. Compared with live perfusion, tumor perfusion was less both before and after treatment (*P < 0.05). ADC_diff_ (b) slightly increased at 12 h suggestive of CA4P-induced intratumoral necrosis, while liver ADC_diff_ did not show significant change. (**D**) Quantification of DCE-MRI biomarkers. Quantitative parameter, percentile value of volume transfer constant K^trans^ (a, *P < 0.05) as well as semi-quantitative parameters including percentile values of area under curve AUC30 (b, ***P < 0.001) and maximal initial slope IS_peak_ (c) and time to peak TTP (d), consistently reflected a reduction of blood flow in necrotic microcancers at 12 h.

The early dramatic reactions in hepatic microcancers could be detected by real-time multiparametric MRI, as demonstrated by a representative hepatic microcancer Tumor 1 (Figure 3A). At baseline, the microcancer appeared slightly hyperintense on T2WI (Figure 3Aa1), nearly isointensity on T1WI (Figure 3Aa4) (Figure 3Aa2), moderately hyperintense on ADC map (Figure 3Aa3) and nearly unenhanced on CE-T1WI, suggestive of intrinsic hypovascularity. Twelve hours after CA4P treatment, massive tumoral necrosis was induced, revealed by the strong hyperintensity within entire tumor on T2WI (Figure 3Ab1), increased tumor ADC (Figure 3Ab3), and delayed contrast enhancement on CE-T1WI (Figure 3Ab4) compared with the precontrast T1WI (Figure 3Ab2). These imaging findings were validated by post-mortem microangiographic and histopathologic assessments (Figure 3B). Microangiography depicted the reduced tumor vessel density (Figure 3Ba). Gross specimen of tumor-bearing liver tissue (Figure 3Bb) and corresponding H&E stained photomicrograph confirmed the nearly complete tumoral necrosis superimposing on the cirrhotic liver (Figure 3Bc1, 3Bc2).

### Blood perfusion drop in cirrhotic liver attributable to massive necrosis in microcancer?

Given the general consensus that small tumors tend to poorly respond to VDA treatment due to the lack of their own established vasculature [18], we next examined our hypothesis that the blood supply in the surrounding cirrhotic liver had been lowered by CA4P as well and, therefore, it caused secondary necrosis in microcancers that are more vulnerable than cirrhotic liver to ischemia. Vascular behaviors both in the tumor and the surrounding cirrhotic liver were assessed by ADC calculations. Quantitative ADC_perf_ indicated that, first at baseline, cirrhotic liver appears significantly hyperperfused relative to micro-HCCs (*p* < 0.05); secondly, CA4P sharply decreased blood perfusion in the surrounding liver (*p* < 0.05) with consequent drop of tumor perfusion (Figure 3Ca). This finding suggests that vasculature of cirrhotic liver was also severely targeted by CA4P, resulting in continuous liver ischemia for at least 12 h and a secondary damage to hepatic microcancers that totally rely on the supply from the surrounding liver. Meanwhile, tumor ADC_diff_ slightly rose, but was nearly unchanged in the surrounding liver, suggestive of tumoral necrosis formation (Figure 3Cb). Furthermore, necrosis-related drop of tumor blood flow from intravascular into extravascular extracellular space (EES) was also demonstrated by multiple DCE parameters, including significantly lowered K^trans^ and AUC30, descending trend of IS_peak_, and upward trend of TTP at 12 h (Figure 3D).

### Massive necrosis in hepatic microcancer along with scattered necrosis in cirrhosis liver

**Figure 4:**
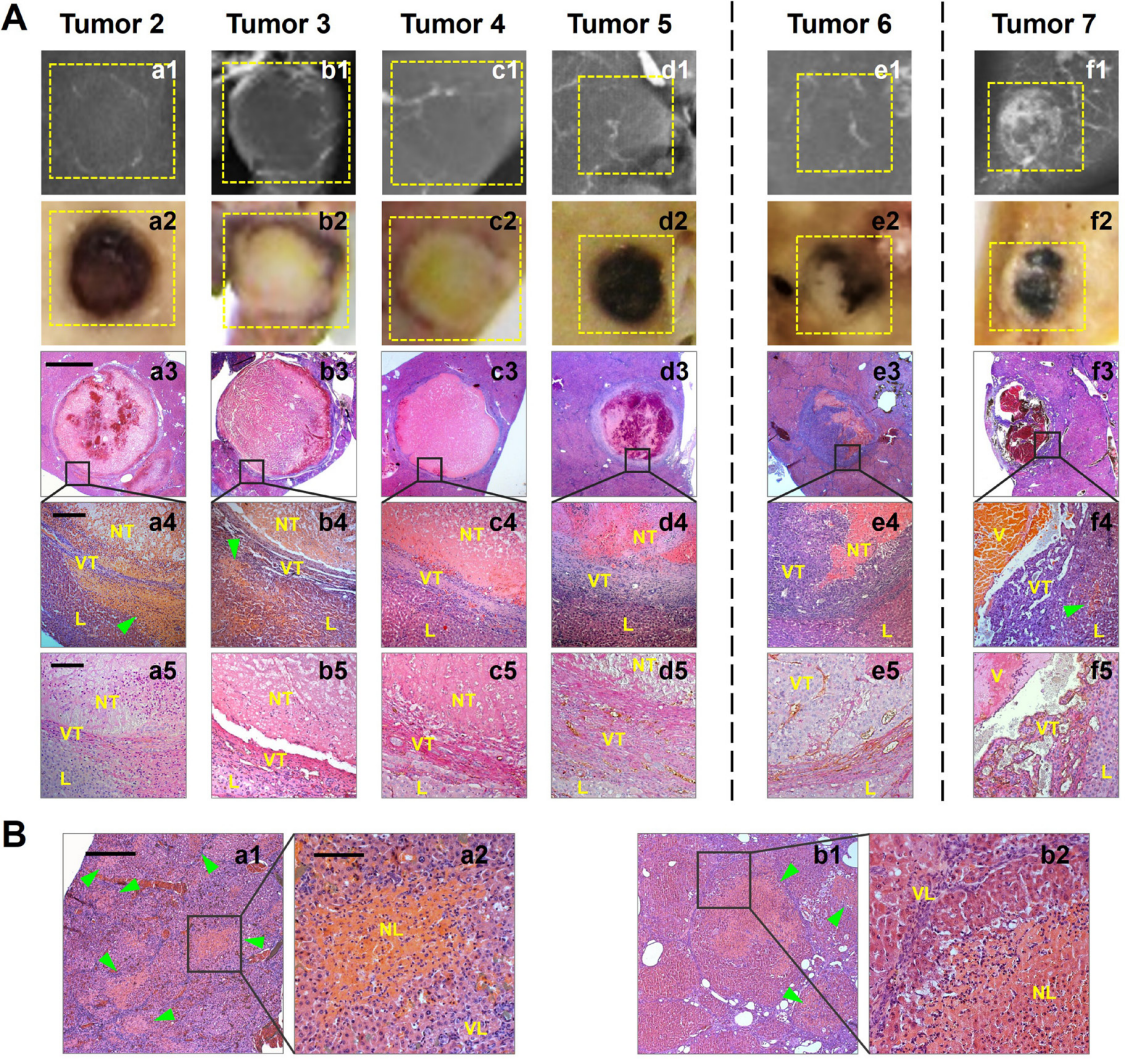
Post-morterm verifications of CA4P-induced necrosis in micro-HCCs on liver cirrhosis (**A**) Microangiography (a1-f1) suggested sparser vessel density appeared in Tumor 2–6 where tumoral necrosis occurred, and large vascular lakes existed in Tumor 7 where rare tumoral necrosis was induced. Photomacrographs (a2-f2) and microscopies (H&E staining; a3-f3, × 12.5 original magnification, scale bar = 800 μm; a4-f4, × 100 original magnification, scale bar = 100 μm.) verified nearly complete necrosis occurring in Tumor 2–5, partial necrosis induced in Tumor 6, and rare necrosis in Tumor 7 (NT: necrotic tumor; VT: viable tumor; L: liver; V: vascular lake.). Patchy necrosis (arrowheads) was also scattered in the sounding cirrhotic liver of Tumor 2, 3 and 7. CD34-PAS dual staining (a5-f5, × 200 original magnification, scale bar = 50 μm) revealed rare CD34-positive vascular channels in the acute necrotic areas in Tumor 2–5, abundant CD34-positive mature vessels in the viable area of Tumor 6 that survived CA4P treatment, and strong and diffuse CD34 expression in tumor cells that lined up the enlarged vascular lakes in Tumor 7. (**B**) Histopathology (H&E staining; a1, b1, × 50 original magnification, scale bar = 200 μm; a2, b2, × 200 original magnification, scale bar = 50 μm.) demonstrated necrosis foci (arrowheads) existed in the cirrhotic liver psarenchyma (NL: necrotic liver; VL: viable liver.).

By histopathologic and microangiographic analyses, the tumor reactions to CA4P therapy were further compared in all those 7 microcancers Table 1. Consequently, nearly complete necrosis (82.8–96.5%) was induced in Tumor 1–5 of largely hypovascular and better differentiated (Grade I–III) micro-HCCs. (Figure 3B; Figure 4Aa1–a4, 4Ab1–b4, 4Ac1–c4, 4Ad1–d4); while partial tumoral necrosis (23.2%) was seen in the hypervascular Grade III poorly differentiated Tumor 6 (Figure 4Ae1–e4) and minimal necrosis in Grade IV undifferentiated Tumor 7 that was composed largely with vascular lakes (Figure 4Af1–f4). To compare tumoral vascularity in microcancers with different therapeutic responses, CD34-PAS dual staining were used. In nearly completely necrotic micro-HCCs, rare CD34-positive channels were shown in the acute central necrosis, indicative of intrinsic hypovascularity (Figure 4a5–d5). However, in largely survived Tumor 6, CD34-positive capillaries were frequently observed, indicative of partial hypervascularity (Figure 4e5); while in Tumor 7, CD34 were diffusely expressed in the tumor cells that lined up the vascular lakes, indicative of vascular lakes and rich tumoral blood supply (Figure 4f5). On top of that, necrotic foci scattered in the cirrhotic liver as seen by microscopy suggested neovasculature in cirrhotic parenchyma could also be attacked by CA4P (Figure 4B).

## DISCUSSION

The development of HCCs in the cirrhotic liver is characterized by multistep remodeling of tumor blood supply [24]. HCCs are generally hypervascularized solid tumors [24] that are fed predominantly by hepatic arterial branches [25]. But, small HCCs in human patients (< 2 cm) are frequently not hypervascular [26] and can be further divided into two types, namely vaguely nodular, well-differentiated tumor (“early” HCC), and distinctly nodular lesion with similar histological feature to “classic” large HCC (small HCC of distinctly nodular type) [27]. Likewise, in DENA-induced rat liver cancer, it has been shown that hepatic tumor nodules smaller than 5 mm are fundamentally supplied by the portal vein, which is distinguishing from the large rat HCCs [20]. Given the diverse HCC vascularity, it would be valuable to analyze the early HCCs as a subgroup of micro-HCCs.

In this preclinical study, we reported for the first time that nearly complete CA4P therapeutic responses was found in certain microcancers (<5mm) of DENA-induced primary HCCs in rats, along with treatment-induced necrotic foci scattered on the cirrhotic liver background. Perfusion and diffusion calculated from ADC, as well as quantitative and semi-quantitative parameters derived from DCE-MRI, helped to portray early tumoral vascular reaction and necrosis, secondary to the dramatic fall of entire liver blood supply. Two main factors may synergistically contribute to this superior efficacy of CA4P in microcancers, 1) avascular and/or hypovascular features in such “early HCCs”, “small HCCs” or micro-HCCs [17, 18, 26]; and 2) neovascularization in cirrhotic liver parenchyma also vulnerable to CA4P mediated antitubulin effects. Consequently, vascular shutdown and ischemic necrosis in the host liver could render the micro-HCCs deprived from vital nutrition, leading to the present paradoxical findings.

Liver cirrhosis has been widely considered as a high-risk precancerous condition which could be due to chronic viral hepatitis, alcohol, aflatoxin, etc. [28, 29]. Application of carcinogen DENA in rodents could simulate this pathological progression and eventually induces primary liver cancers with underlying liver cirrhosis [30]. Development of liver fibrosis is associated with pathological angiogenesis that progressively forms the abnormal angioarchitecture distinctive of cirrhotic liver [31, 32]. Notably, CA4P-induced necrosis in cirrhotic liver as seen in our study seems to suggest that neo-angiogenesis in liver fibrogenic progress might share something in common with tumor angiogenesis that can be equally targeted by VDAs. However, the mechanisms remain to be explored.

We recognize the limitations of our study. Although DWI has been a renowned imaging marker to monitor early VDA-induced tumor vascular responses and further indicate tumoral necrosis [10, 23], the calculated ADC changes concerning tumor perfusion and diffusion in our study did not appear so significantly. The associated reasons might be the increased MRI artifact when tumor volume is too small, and the limited cases of recruited hepatic microcancers in this study. Secondly, considering the significant alterations of blood supply in the cirrhotic liver and the lack of reports regarding such side effect of VDA in liver cirrhosis in the clinical studies, it would be valuable to conduct parallel studies to compare VDA effects in normal and cirrhotic livers. In addition, the body size disparity between human and rodent may arouse suspicion about the relevance between these rat-specific findings and the similar effects in human patients, which calls for further validation in the human clinical trials.

## MATERIALS AND METHODS

### Animals and reagents

Male Sprague Dawley (SD) rats were purchased from Charles River Breeding Laboratories, Inc. (St. Aubain les Elbeuf, France). Diethylnitrosamine (DENA, N0258) was procured from Sigma-Aldrich (St. Louis, MO, USA). CA4P (C643025) was obtained from Toronto Research Chemical Inc. (Toronto, Canada). MRI contrast agent Dotarem (Gd-DOTA, Gadoterate meglumine; Dotarem^®^, Guerbet, France), barium sulfate suspension (Micropaque^®^, Guerbet, France) and gas anesthetic isoflurane (Forane^®^; Baxter Healthcare, Deerfield, IL, USA) were also commercially obtained. The rabbit monoclonal antibody against CD34 was purchased from Abcam (ab62738).

### *In vivo* MRI

Images were acquired on a clinical 3.0T MRI scanner (MAGNETOM Prisma; Siemens, Erlangen, Germany) and a human wrist coil (Hand/Wrist 16, A 3T Tim coil, Siemens). Twenty axial images were acquired, with a slice thickness of 2.0 mm and a gap of 0.4 mm. T2-weighted (repetition time, 4000 ms; echo time, 70 ms; flip angle, 150°; field of view, 75 × 56 mm^2^; matrix, 256 × 192) and T1-weighted (repetition time, 626 ms; echo time, 15 ms; flip angle, 160°; field of view, 75 × 56 mm^2^; matrix, 256 × 192) turbo spin echo turbo spin echo (TSE) images (T2WI, T1WI) were performed weekly to monitor tumor growth, while T2WI, T1WI, DWI, DCE and consecutive contrast-enhanced (CE)-T1WIs were acquired to evaluate CA4P treatment. For DWI, a 2-dimensional SE echo-planar imaging (EPI) sequence (repetition time, 3500 ms; echo time, 62 ms; flip angle, 90°; field of view, 136 × 74 mm^2^; matrix, 96 × 52) with 8 b values (0, 50, 100, 150, 400, 600, 800 and 1000 seconds/mm^2^) was acquired. For DCE, a T1-weighted gradient echo (GE) sequence (repetition time, 7 ms; echo time, 2.45 ms; flip angle, 15°; field of view, 61 × 89 mm^2^; matrix, 132 × 192) was acquired. After a precontrast baseline of 30 measurements, a bolus injection of 0.02 mmol/kg Gd-DOTA was conducted. The entire acquisition lasted for 100th measurement. Immediately after DCE, a bolus of 0.2 mmol/kg Gd-DOTA was injected before a series of CE-T1WIs were acquired.

### Experimental design

This animal experiment was carried out in compliance with European and national regulations after approval from KU Leuven university ethics committee for animal care and use. All *in vivo* procedures including gavage feeding, tumor implantation, drug injection and imaging were performed under gas-anesthesia with 2% isoflurane in the mixture of 20% oxygen and 80% room air using a gas anesthesia system (Harvard Apparatus, Holliston, MA, USA).

As illustrated in Figure 1, multifocal primary liver cancers were established in 19 male Sprague Dawley (SD) rats weighting 300–350 g by 8-week daily gavage feeding of DENA at 10 mg/kg/day. Tumor growth was monitored weekly by MRI from the 9th week after DENA administration until the largest tumor lesion attained more than 1 cm in diameter. All recruited tumor-carrying rats received single intravenous injection of CA4P at 10 mg/kg. T2WI, T1WI, DWI, DCE and CE-T1WI were performed 4 h before and 12 h after CA4P therapy. Rats were euthanized after the last time point of MRI for postmortem microangiography and histopathology.

### MR image analyses

Image analysis was conducted using the built-in software on the Siemens workstation (version Numaris/4 Syngo MR A30), MeVisLab (version 2.6.2, MeVis Medical Solutions AG, Bremen, Germany) and MatLab (version R2015b, The MathWorks, Natick, MA, USA). All the following measurements were acquired by 3 authors with consensus.

#### Measurement of tumor diameter

On T2WI, tumor diameter was manually measured from the tumor-containing image with the largest tumor cross section at 4 h before treatment.

#### Separate calculation of tumor ADCs

On DWI, tumor area was manually contoured with an operator-defined region of interest (ROI) on all tumor-containing images. ADC map was derived from DWI according to the following mono-exponential formula: S_i_ = S0 × exp (-b_i_ × ADC), in which Si is the signal intensity (SI) measured on the _i_th b value image, bi is the corresponding b value, and S_0_ is a variable estimating the intrinsic SI (for b = 0 seconds/mm^2^).

For the calculation of different ADC values, microcancers were freehand delineated only on the central slices with the largest cross-sectional areas on the original DWIs at the b value of 1000 s/mm^2^, in order to avoid partial volume effects. The delineations of each microcancer lesion and liver were copied to all images with different b values automatically. The average SI per tumor and per b value was then determined. The difference between ADC_low_ (b = 0, 50 and 100 s/mm^2^) and ADC_high_ (b = 400, 600 and 1000 s/mm^2^) was defined as ADC_perf_ to reflect the tissue micro-vessel or capillary perfusion, while ADC_high_ was defined as ADC_diff_ [33].

#### Quantitative and semi-quantitative analyses of T1-weighted DCE

For the calculation of multiple DCE parameters, ROI of tumor was freehand delineated on the central slices with the largest cross-sectional areas; ROI of abdominal aorta was manually delineated from 4 consecutive slices for defining arterial input function; ROI of cirrhotic liver was manually delineated on 4 representative slices each from median, left, right and caudate lobes. All ROIs were copied to all measurements automatically. Since a low gadolinium dose was used, a linear relation between the amount of contrast agent in the tissue and the resultant difference in relaxation time could been assumed [34]. Quantitative parameter K^trans^, the volume transfer constant between blood plasma and EES, was generated by fitting Tofts and Kermode model [35, 36]. Semi-quantitative parameters, such as area under the time-signal intensity curve (AUC30), maximal initial slope (ISpeak) and time to peak (TTP) were derived from the enhancement curve [37].

### Digital microangiography

After the last MRI scanning, rats were anesthetized by an intraperitoneal injection of pentobarbital at 50 mg/kg. Then laparotomy was performed with blood collected via postcava and abdominal aorta cannulated, through which barium suspension was injected before the entire tumor-bearing liver was excised. With a digital mammography unit (Em-brace; Agfa-Gevaert, Mortsel, Belgium), postmortem hepatic arteriography was made at 26 kV, 32 mAs to document changes in tumor vascularity. The livers were then fixed and sliced into 3-mm sections in the axial plane corresponding to the MR images, and these sections were radiographed at 26 kV, 18 mAs.

### Histopathological analyses

After microangiography, the tumor sections were paraffin imbedded, sliced into 5 μm thickness and stained with hematoxylin and eosin (H&E) for microscopic analysis using an Axiovert 200 M microscope equipped with an AxioCam MR monochrome digital camera (Carl Zeiss Inc, Gottingen, Germany) and by AxioVision 4.8 software.

#### Diagnosis of HCCs

Due to the high analogy to the histopathological progression observed in human liver cancer, rat primary HCCs were diagnosed according to the classical histomorphologic features: malignant hepatocytic tumors, often well vascularized, with wide trabeculae (>3 cell layers), noticeable acinar pattern, small cell changes, cytologic atypia, prominent nucleoli, mitotic activity, vascular invasion, absence of Kupffer cells, missing portal triads, and the loss of the reticulin network [29]. The differentiation of rat HCCs was further classified into Grade I-IV using a modified 4-scale Edmondson and Steiner system [29]. Tumor vascularity was semi-quantificationally scored from + to ++++ (Table 1).

#### Calculation of CA4P-induced intratumoral necrosis

Digital images of tumor slices at a magnification of 12.5 were used to estimate the percentage of tumor necrosis by ImageJ software [38]. Briefly, regions of interest were delineated around the entire tumor and the necrotic tumor, respectively, to get ‘necrotic ratio on each section’. For each tumor section, the axial slide representing this tumor block was selected as ‘section area’. Tumor necrosis on each H&E stained slice was estimated independently by 2 pathologists, and calculated with the equation: Intratumoral necrosis ratio (%) = ∑ [Necrotic ratio on each section (%) × section area (mm^2^)] × section thickness (mm) / [4/3π r^3^] (mm^3^).

### CD34 and periodic acid-Schiff (PAS) dual staining

To identify matrix-associated vascular channels of HCCs, CD34 immunohistochemical staining was first performed. Paraffin-embedded slices in 5 μm thickness were deparaffinized, rinsed and exposed to 3% H_2_O_2_ for 10 min at 37°C to block the activity of endogenous peroxidase. The slices were then blocked with 10% goat serum for 30 min at room temperature, before incubation with a CD34 antibody at a dilution of 1:150 overnight at 4°C. The reactions were visualized with the Histostain-SP-Broad-Spectrum kit (Invitrogen, Grand Island, NY, USA). Following CD34 immunostaining, slices were rinsed and treated with 0.5% PAS (American MasterTech, CA, USA) for 15 min, counterstained with hematoxylin and cover-slipped.

### Statistical analysis

Statistical analyses were carried out by GraphPad Prism (version 7.02, GraphPad Software Inc, La Jolla, CA, USA). The Pearson's correlation coefficient was calculated between percentile tumoral necrosis calculated by histopathology and tumor diameter measured from T2WI. Numerical data were presented as mean ± standard errors of the mean (SEM) or standard deviation (SD). Comparison of percentile tumoral necrosis and DCE parameters were performed by unpaired two-way *t*-test; results of ADCs between tumor and liver background were compared by two-way ANOVA. A significant difference was concluded for *P* < 0.05.

## CONCLUSIONS

On balance, the present findings may shed some light on the preventative effect of CA4P on recurrent hepatoma foci as well as intrahepatic micrometastases in cirrhotic background. But on the other hand, such a phenomenon also raise the awareness to protect liver function during future CA4P therapy principally in patients with underlying chronic hepatic diseases being developed into cirrhosis, for potential formation of CA4P-induced necrosis in cirrhotic liver and consequent liver failure. These may be of potential value for planning further clinical applications of CA4P in human subjects with HCCs and liver cirrhosis.

## Abbreviations

ADC: apparent diffusion coefficient
CA4P: combretastatin A-4 phosphate
CE: contrast-enhanced
DCE: dynamic contrast enhanced
DENA: diethylnitrosamine
DWI: diffusion-weighted imaging
EES: extracellular extravascular space
EPI: echo-planar imaging
GE: gradient echo
H&E: hematoxylin and eosin
HCC: hepatocellular carcinoma
MRI: magnetic resonance imaging
PAS: periodic acid-Schiff
RECIST: Response Evaluation Criteria in Solid Tumors
ROI: region of interest
SD: Sprague Dawley
SEM: standard errors of the mean
SI: signal intensity
T1WI: T1-weighted imaging
T2WI: T2-weighted imaging
TSE: turbo spin echo
VDA: vascular disrupting agent.
